# Early protein intake predicts functional connectivity and neurocognition in preterm born children

**DOI:** 10.1038/s41598-021-83125-z

**Published:** 2021-02-18

**Authors:** Emma G. Duerden, Benjamin Thompson, Tanya Poppe, Jane Alsweiler, Greg Gamble, Yannan Jiang, Myra Leung, Anna C. Tottman, Trecia Wouldes, Steven P. Miller, Jane E. Harding, PIANO study group, Jane M. Alsweiler, Janene B. Biggs, Coila Bevan, Joanna M. Black, Frank H. Bloomfield, Kelly Fredell, Greg D. Gamble, Jane E. Harding, Sabine Huth, Yannan Jiang, Christine Kevan, Myra Leung, Geraint Phillips, Tanya Poppe, Jennifer A. Rogers, Heather Stewart, Benjamin S. Thompson, Anna C. Tottman, Kathryn A. Williamson, Trecia A. Wouldes

**Affiliations:** 1 Applied Psychology, Faculty of Education, Western University, London, ON Canada; 2 Department of Paediatrics, The Hospital for Sick Children, University of Toronto, Toronto, ON Canada; 3 School of Optometry and Vision Science, University of Waterloo, Waterloo, Canada; 4 School of Optometry and Vision Science, University of Auckland, Auckland, New Zealand; 5 Liggins Institute, University of Auckland, Auckland, New Zealand; 6 Department of Paediatrics: Child and Youth Health, University of Auckland, Auckland, New Zealand; 7 Department of Psychological Medicine, University of Auckland, Auckland, New Zealand; 8 Centre for the Developing Brain, King’s College London, London, United Kingdom

**Keywords:** Outcomes research, Paediatric research, Computational neuroscience, Cognitive neuroscience, Development of the nervous system

## Abstract

Nutritional intake can promote early neonatal brain development in very preterm born neonates (< 32 weeks’ gestation). In a group of 7-year-old very preterm born children followed since birth, we examined whether early nutrient intake in the first weeks of life would be associated with long-term brain function and neurocognitive skills at school age. Children underwent resting-state functional MRI (fMRI), intelligence testing (Wechsler Intelligence Scale for Children, 5th Ed) and visual-motor processing (Beery-Buktenica, 5th Ed) at 7 years. Relationships were assessed between neonatal macronutrient intakes, functional connectivity strength between thalamic and default mode networks (DMN), and neuro-cognitive function using multivariable regression. Greater functional connectivity strength between thalamic networks and DMN was associated with greater intake of protein in the first week (β = 0.17; 95% CI 0.11, 0.23, p < 0.001) but lower intakes of fat (β = − 0.06; 95% CI − 0.09, − 0.02, p = 0.001) and carbohydrates (β = − 0.03; 95% CI − 0.04, − 0.01, p = 0.003). Connectivity strength was also associated with protein intake during the first month (β = 0.22; 95% CI 0.06, 0.37, p = 0.006). Importantly, greater thalamic-DMN connectivity strength was associated with higher processing speed indices (β = 26.9; 95% CI 4.21, 49.49, p = 0.02) and visual processing scores (β = 9.03; 95% CI 2.27, 15.79, p = 0.009). Optimizing early protein intake may contribute to promoting long-term brain health in preterm-born children.

## Introduction

Very preterm birth (< 32 weeks’ gestational age) remains a significant predictor of morbidity, with a sizable proportion of survivors developing motor difficulties^1,2^. Neonates born at younger gestational ages are at a higher risk of adverse outcomes^3^, and the risk is highest in those born < 30 weeks’ gestation^4,5^. Those neonates who escape major deficits may also go on to have subtle deficits in cognitive ability that are revealed at preschool and school age^6,7^. Cognitive and motor impairments in children born preterm have been associated with brain dysmaturation, particularly in the prefrontal cortices, which are essential for higher order cognitive functions. Further, longitudinal and cross-sectional neuroimaging studies have reported alterations in brain structural and functional development in infants, children and adolescents born preterm, including alterations in white matter^8^, decreases in brain volumes and cortical thickness in regionally specific locations in the thalamus, amygdala, hippocampus, frontal, parietal and temporal cortices^9–12^, with greater degree of prematurity associated with larger changes^13^.

Resting-state functional MRI has provided insight into functional connectivity patterns in infants and children born preterm^14–16^. Alterations in structural and intrinsic functional connectivity affecting networks associated with higher-order cognitive functions including default mode networks (DMN) have been reported in school-age children, adolescents and adults born preterm^14,17,18^. The DMN develops in the third trimester, which is marked by substantial increases in both functional connectivity and development of white matter supporting a globally integrated network of brain regions^19–21^. The DMN is separated into anterior and posterior subdivisions^22^. The anterior DMN includes portions of the medial and lateral prefrontal cortices as well as the anterior cingulate cortex, and anterior temporal lobe. It is involved in higher order cognitive functions^21^ and early disruptions in this network may underlie cognitive deficits in children born preterm^23,24^.

Some evidence suggests that enhanced nutrition may protect brain structural and functional development in children born preterm^25,26^. For example, protein and energy intake during the first week after birth positively predicted cognitive outcomes at 18 months in a cohort of extremely low birth weight infants^27^. In another cohort of very preterm born infants who underwent serial MRI scans from birth, total energy, carbohydrates, lipids and protein intake in the first few weeks positively predicted larger brain volumes at term equivalent age as well as cognitive outcomes at 18 months^28^. Further, compared to preterm infants randomized to a standard diet, those randomized to a high nutrient diet had larger caudate nucleus volumes in adolescence which correlated with higher verbal IQs, particularly in boys^29^. However, there have been no studies of the relationship between very early macronutrient intakes in very preterm infants and brain connectivity and neurodevelopmental outcomes at school age, when different aspects of brain function can be more reliably assessed.

We examined brain functional connectivity and cognitive and visuomotor ability at 7 years of age in a group of children born very preterm in whom daily actual macronutrient intakes and clinical variables were collected prospectively. During the course of the study, the amount of dietary intake of protein was increased as part of a planned change in clinical care to better meet international recommendations^30^, as previously described^31^. We focused on frontal resting state networks, given previous research suggesting structural and functional alterations in the prefrontal cortices underlying cognitive^32^ and visual perceptual abilities^33^ in children born preterm. Our a-priori hypotheses were that early neonatal macronutrient intakes would be associated with functional connectivity between the thalamic and default mode networks (DMN) at 7 years, and that functional connectivity would be associated with cognitive ability and visual-motor function.

## Methods

### Participants

The PIANO Study cohort has been reported previously^34^. In brief, all infants born at < 30 weeks’ gestation or < 1500 g between July 2005 and October 2008 inclusive and admitted to the neonatal intensive care unit at National Women’s Hospital, Auckland, New Zealand for at least the first postnatal week were eligible for recruitment into a follow-up study at 7 years of age. Of the 128 children assessed at 7 years (28% of original cohort), 113 consented to MRI, 102 completed at least some MRI sequences, and 56 successfully completed resting-state functional MRI sequences. The study was approved by the Northern B ethics committee (NTY/12/05/035) and the Auckland District Health Board (ADHB 5486). A statement to confirm that all methods were carried out in accordance with relevant guidelines and regulations. Informed consent was obtained from a parent and/or legal guardian.

Clinical data were collected from the medical records by trained clinical research staff. All daily actual parenteral and enteral intakes (excluding blood products) were recorded for the first 28 days. Energy intake was computed with proteins and carbohydrates providing 4 kilocalories (kcal) per gram and lipids providing 9 kcal per gram. Breast milk and formula composition was based on commercial notifications^34^. Intakes per kg were calculated for each day using the most recent highest weight, and averaged for the first 7 days and first 28 days (first month).

### Magnetic resonance imaging and analysis

Children underwent anatomical and functional MRI on a 3 T scanner (Siemens, Skyra, Erlangen, Germany). Anatomical images were acquired using an MPRAGE pulse sequence ([repetition time]TR, 2000 ms, [echo time]TE, 3510 ms, [inversion time]TI, 1010 ms, slice thickness, 0.85 mm, FOV, 210 × 210 mm). Resting-state fMRI was acquired using an echo-planar imaging sequence (TR, 3200 ms, TE, 40 ms, FOV, 147 × 147 mm, matrix, 64 × 64, slice thickness, 3 mm).

Resting-state fMRI data were preprocessed using the FMRIB Software Library (FSL; version 5.0.6). Preprocessing included: slice timing and motion correction, spatial smoothing, band-pass filtering (suppressing physiological noise) and whole-brain tissue extraction. Data were nonlinearly registered to a standardized MRI template in MNI (Montreal Neurological Institute) space. Whole-brain resting state connectivity was examined using a group temporal concatenation independent components analysis (ICA), available in FSL using MELODIC (Multivariate Exploratory Linear Decomposition into Independent Components, version 4.0). This produced a set of independent components common to the whole group. All resting-state components were visually inspected. Resulting ICA maps were thresholded at Z ≥ 4. The time courses from the independent components were extracted.

### Neuropsychological testing

Children underwent neuropsychological testing by a trained assessor. Full scale IQ (FSIQ) was assessed using the Wechsler Intelligence Scale for Children 5th Ed (WISC-V) (standard mean score 100, standard deviation [SD] 15)^29^. Subtests include the perceptual reasoning index (PRI), processing speed index (PSI), working memory index (WMI), and the verbal comprehension index (VCI).

Visuomotor integration was assessed using Beery–Buktenica Developmental Test of Visual–Motor Integration (Beery), 5th edition^35^, which assesses motor and perceptual skills. The maximum raw score for each of the three sub-tests is 30. The scores were converted to standardized scores.

### Statistical analysis

All analyses were carried out using Statistical Package for the Social Sciences (v.25 SPSS, Chicago, IL). The time courses from the thalamic and DMN were extracted. Correlation coefficients describing the connectivity strength between the thalamus and the DMN time courses were calculated for each participant. To assess the relationship between early macronutrient intakes and functional connectivity strength between the subcortical and cortical resting-state networks, we used a general linear model, with the correlation coefficients describing thalamocortical connectivity strength entered as the dependent variables and macronutrient intakes (i.e., protein, carbohydrates, fats, total energy) as independent variables. Analyses were adjusted for gestational age (GA) at birth, birth weight z score, age at scan and biological sex. Birthweight z score reflects growth before birth, and is expressed in standard deviation scores, which are independent of absolute weight. Since we had one *a-priori* hypothesis (that neonatal macronutrient intake would be associated with functional connectivity between the thalamic and cortical networks at 7 years of age), we set the alpha level at 0.05.

To assess the relationships between functional connectivity strength and 7-year cognitive and visuomotor abilities, we used general linear models, with the correlation coefficients describing the connectivity strength between the thalamic and cortical networks entered as independent variables and the scores on the WISC-V subtests and the Beery entered as dependent variables. Analyses were adjusted for GA at birth, birth weight z score, age at scan and biological sex. Since we had two a-priori hypotheses (that both cognitive and visuomotor scores would be associated with functional connectivity at 7 years), we set the alpha level at 0.025.

## Results

Of the total 113 children, 102 children underwent MRI, of whom 13 were unable to complete the resting-state scans, and an additional 33 children moved excessively during the scans. One of these children had grade III/IV IVH as a neonate. Analyzable data were available for 56 children (Table 1). The neonatal macronutrient intake for the cohort is available in Table 2. No significant differences in sex (p = 0.30), adjusted age (t = − 0.9, p = 0.35), GA at birth (t = 0.22, p = 0.08), or birth weight (t = 0.91, p = 0.37) were evident between the children with useable MRI data compared to the children in the full cohort. There were also no differences in neonatal macronutrient intakes between the children with included MRI data and the other children in the full cohort (all p > 0.05).

**Table 1:** Participant demographics.

	n = 56
Males (%)	26 (46)
Birth GA, weeks, median (IQR)	26 (25–28)
Birth weight, grams, median (IQR)	853 g (770–1030)
IVH grade III/IV (%)	2 (4)
Age, years, median (IQR)	7.5 (7.4–7.6)

Demographics and clinical variables of participants who completed the MRI scans.

*GA* gestational age, *IQR* interquartile range, *IVH* intraventricular hemorrhage.

**Table 2:** Neonatal macronutrient intake.

	Medians [interquartile ranges]
**Macronutrients—first 7 days**	
Protein, g/kg/day	2.38 [2.1–2.7]
Carbohydrates, g/kg/day	10.71 [9.24–12.19]
Fats, g/kg/day	3.74 [3.02–4.21]
Energy, kcal/kg/day	83.25 [77.49–91.71]
**Macronutrients—first 28 days**	
Protein, g/kg/day	3.41 [3.14–3.53]
Carbohydrates, g/kg/day	14.89 [13.88–15.90]
Fats, g/kg/day	6.19 [5.66–6.52]
Energy, kcal/kg/day	128.94 [119.97–133.82]

### Cognitive outcomes

The majority of children performed within the typical range (i.e., 70–130) on the WISC-V (Table 3). GA at birth was related to FSIQ, VCI, PRI, and PSI scores (all, p < 0.05), but sex was not related to any of the WISC-V scores (p > 0.33). No significant differences were noted between the children whose MRI data were or were not included on the WISC-V subtests (all, p > 0.05), except for the PSI (p = 0.04).

**Table 3:** Medians and interquartile ranges for the cognitive and visuomotor outcomes.

**WISC-V**	
FSIQ	95 (86–104)
VCI	98 (90–106)
WMI	94 (84–102)
PSI	97 (88–106)
PRI	90 (80–102)
**Beery**	
Visuomotor integration	94 (88–99)
Visual perception	101 (89–115)
Motor ability	92 (83–102)

*WISC-V* Wechsler Intelligence Scale for Children, fifth edition, *FSIQ* full scale intelligence quotient, *VCI* verbal comprehension index, *WMI* working memory index, *PSI* processing speed index, *PRI* perceptual reasoning index, *Beery* Beery-Buktenica developmental test of visual-motor integration, 5th edition.

### Visuomotor outcomes

Most scores on the Beery were also in the typical range (Table 3). GA was not associated with any of the visuomotor outcome scores (all, p > 0.06), but girls had higher scores than boys (B = 0.21 [females], p = 0.003). No significant differences were noted between the children whose MRI data were or were not included on the Beery (all, p > 0.05).

### Resting-state networks

**Figure 1:**
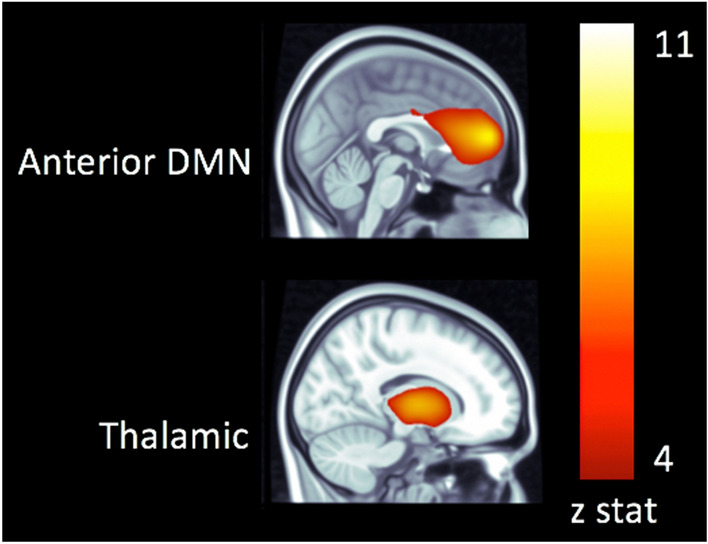
Resting state networks overlaid on a template MRI in the space of the Montreal Neurological Institute.

Independent components analysis produced whole-brain functional connectivity maps, with robust resting state activity in anterior default mode (aDMN) and thalamic networks (Fig. 1).

**Figure 2:**
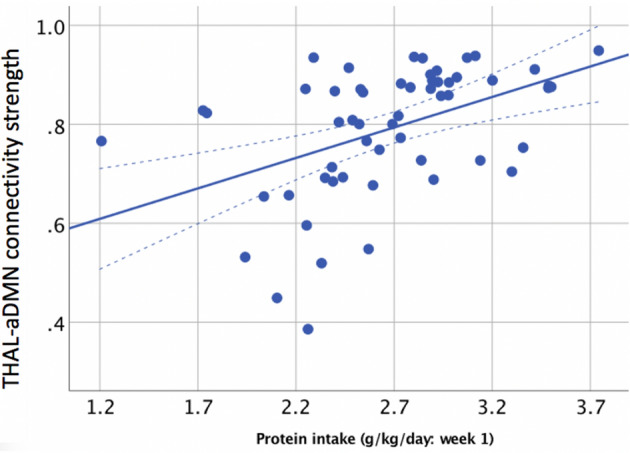
Relationship between functional connectivity strength in the thalamic and aDM resting state networks at 7 years (y-axis) and protein intake in the first week after birth (x-axis).

Greater functional connectivity strength between the thalamic networks and aDMN (range: 0–1) was predicted by greater intake of protein in the first week (β = 0.17; 95% CI 0.11, 0.23, p < 0.001, Fig. 2) but lower intakes of fat (β = − 0.06; 95% CI − 0.09, − 0.02, p = 0.001) and carbohydrates (β = − 0.03; 95% CI − 0.04, − 0.01, p = 0.003). Total energy intake in the first week did not predict thalamocortical connectivity strength (β = -0.002; 95% CI − 0.006, 0.001, p = 0.1). Thalamocortical connectivity strength was also positively associated with increased intake of protein in the first 28 days (β = 0.22; 95% CI 0.06, 0.37, p = 0.006) but not with intakes of fats, carbohydrates or total energy (all p > 0.1).

**Figure 3:**
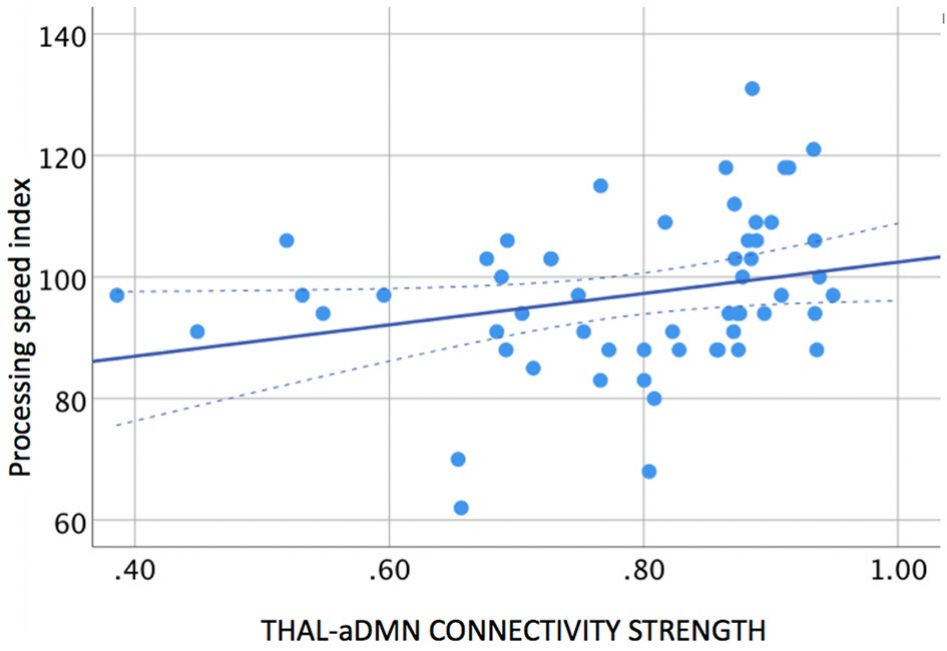
Relationship between functional connectivity strength in the thalamic and aDM resting state networks (x-axis) and processing speed index (y-axis) at 7 years.

In turn, thalamocortical connectivity strength predicted higher scores for the PSI subtest of the WISC (β = 26.9; 95% CI 4.21, 49.49, p = 0.02, Fig. 3) but not for the WMI (β = 23.46; 95% CI 0.001, 46.9, p = 0.0.5), PRI (β = 23.25; 95% CI − 3.14, 49.64, p = 0.0.8), or VCI subtest scores (β = − 3.1; 95% CI − 29.91, 20.67, p = 0.8) or full-scale IQ (β = 20.2; 95% CI − 3.79, 44.18, p = 0.1).

Thalamocortical connectivity strength was also related to visual perceptual scores on the Beery (β = 9.03; 95% CI 2.27, 15.79, p = 0.009), but not the Beery visuomotor index (β = − 2.39; 95% CI − 6.47, 1.7, p = 0.3) or motor scores (β = 2.52; 95% CI − 2.62, 7.66, p = 0.3).

## Discussion

Seven-year-old children born very preterm, who had higher protein intake in the first week and first 28 days after birth positively predicted the strength of the connectivity between subcortical and cortical resting state networks at 7 years of age, and in turn, that this connectivity is associated with specific neurocognitive skills such as processing speed index. Our findings are in line with a growing body of evidence indicating that very early macronutrient intake has long-term effects on preterm brain development and functional outcomes at school age.

### Early nutrients and outcomes in preterm infants

Our observation of an association between early protein intake and functional connectivity between the thalamic and the anterior default mode networks at 7 years of age is consistent with previous reports that nutrition is an important predictor of growth and outcome in preterm neonates^36^. Greater energy and lipid intake during the first two weeks after birth predicted larger subcortical structures, cerebellum, and total brain, and accelerated white matter microstructural maturation over the course of neonatal intensive care to term age^28^.

Protein intake in the first two weeks also predicted 18-month cognitive and motor outcomes in very low birth weight neonates born preterm^37^. Stephens et al. reported that a 1 g/kg/day increase in protein intake up to 2.5–3.5 g/kg/day was independently associated with an ~ 8 point increase in cognitive scores at 18 months^27^. Protein intakes in our study cohort were largely within this range. In contrast to these findings, another report noted that high protein intake (4 g/kg/day) was associated with neurodevelopmental impairments at 2 years in very low birth weight children^38^. These findings suggest that adequate early protein intake is essential for optimal brain growth, although adverse effects of protein overfeeding cannot be excluded. The specific mechanisms regarding early protein intake and the association with functional brain connectivity may be attributed to regional increases in brain growth, promotion of synaptic connectivity or a combination of macrostructural and functional changes that are supportive of neuroplasticity^25^.

Previous studies have reported that early intake of fat and energy rather than protein were important predictors of brain maturation^16^ and later cognitive function^28,39^. However, other nutrients including protein, choline and zinc are also essential for healthy brain development^40^. In our study only protein intake positively predicted 7-year thalamocortical connectivity. Protein is essential for cell proliferation and synaptogenesis, which may support developing thalamocortical connectivity^41^, perhaps by supporting myelination of anterior white matter pathways in the preterm neonatal brain and hence connectivity between thalamic networks and the anterior DMN. Compared with previous studies, our cohort had on average greater energy and fat intakes, and similar or greater protein intakes. Thus, it is possible that protein intake has an additional effect on brain development only if energy intake is adequate. Remarkably, these relationships are seen following nutritional differences even in the first week after birth, emphasising the importance of very early nutrition for optimising long-term outcomes.

### Functional connectivity, cognitive and visuomotor abilities

Resting-state networks have been reliably detected in neonates, infants and children born preterm during the first few weeks to years of life^42,43^. Previous research has demonstrated alterations in thalamic networks and thalamocortical connectivity strength in preterm infants^16,44,45^ and that thalamocortical connectivity predicted cognitive scores in the second year of life^45^. Our findings suggest that alterations in thalamocortical connectivity persist beyond the neonatal and early childhood period and have long lasting effects on brain function and specific cognitive skills. Indeed, the association of functional connectivity with visual perceptual abilities is consistent with previous functional neuroimaging studies of school-age children born preterm showing that altered functional connectivity in the prefrontal cortices is associated with visual-perceptual impairments^33,46^. Our results suggest that alterations in corticothalamic connectivity may contribute to higher order vision deficits associated with preterm birth.

## Conclusions

In a cohort of school aged children born very preterm, greater protein intake during the first week and the first month of life were positively associated with more robust functional connectivity strength between thalamic networks and anterior DMN essential for higher order cognitive functioning. Further, greater strength between the thalamocortical networks was associated with processing speed and visual perceptual abilities at 7 years of age. However, the generalizability of findings from this small study requires confirmation. The full cohort recruited at birth were not assessed with MRI at 7 years. The subset of children included in the current study may not be entirely be representative of the full sample. Future studies with larger samples will permit the examination of early nutrient intake on the development of functional connectivity networks, and also the possible effects of social adversity. Early neonatal nutrition, particularly increasing early protein intake in very preterm neonates, may help mitigate the adverse effects of prematurity and neonatal intensive care on brain development.
